# Levonorgestrel Inhibits Human Endometrial Cell Proliferation through the Upregulation of Gap Junctional Intercellular Communication via the Nuclear Translocation of Ser255 Phosphorylated Cx43

**DOI:** 10.1155/2015/758684

**Published:** 2015-06-16

**Authors:** Xiaomiao Zhao, Xueliang Tang, Tingting Ma, Miao Ding, Lijuan Bian, Dongmei Chen, Yangzhi Li, Liangan Wang, Yanyan Zhuang, Meiqing Xie, Dongzi Yang

**Affiliations:** ^1^Department of Obstetrics and Gynecology, Sun Yat-Sen Memorial Hospital, Sun Yat-Sen University, Guangzhou, Guangdong 510120, China; ^2^Department of Obstetrics and Gynecology, Shenzhen Maternity and Child Care Centers, Southern Medical University, Shenzhen, Guangdong 518000, China; ^3^Pathology Department, Sun Yat-Sen Memorial Hospital, Sun Yat-Sen University, Guangzhou, Guangdong 510120, China; ^4^Department of Obstetrics and Gynecology, The First Affiliated Hospital of Soochow University, Suzhou 215325, China

## Abstract

*Objects.* To assess whether LNG exerts antiproliferation effects on human endometrial cells through changes of GJIC function and the phosphorylated Cx43.* Methods.* Cell proliferation and apoptosis of human endometrial stromal cells (HESCs) and glandular cells (HEGCs) treated with LNG in a dose- and time-dependent manner. GJIC change and further total Cx43 and serine 368 and 255 phosphorylated Cx43 were measured.* Results.* 5 × 10^−5^ mol/L LNG revealed a time-dependent inhibition of cell proliferation and an increase of apoptosis in both HESCs and HEGCs. Furthermore, these cells demonstrated a significant GJIC enhancement upon treatment with 5 × 10^−5^ mol/L for 48 hours. The effects of LNG were most noticeable in HESCs rather than in HEGCs. Associated with these changes, LNG induced a relative increase in total Cx43 in a time-dependent manner but not Ser368 phosphorylated Cx43. Moreover, laser scanning confocal microscope confirmed the increased expression of total Cx43 in the cytoplasm and, interestingly, the nuclear translocation of Ser255 phosphorylated Cx43.* Conclusions*. LNG likely inhibits the proliferation and promotes apoptosis in HESCs and HEGCs though an increase in gap junction permeability in vitro, which is achieved through the upregulation of Cx43 expression and the translocation of serine 255 phosphorylated Cx43 from the plasma to the nuclear compartment.

## 1. Introduction

Endometrial cancer (EC) is the most frequently diagnosed gynecological malignancy, and the incidence is increasing among women with younger and younger age in the last decade, partly owning to the increasing incidence of polycystic ovary syndrome which may cause long-term single estrogen effect in vivo. Endometrial complex atypical hyperplasia (CAH) is a precursor to endometrioid adenocarcinoma, and untreated CAH carries a risk of progression to carcinoma of approximately 25% [1, 2]. Hyperplasic endometrial polyps, in spite of their rare malignant changes, were considered as a risk factor for EC [3, 4]. Recent data from Saccardi et al. showed that there was a strong correlation between previous history of abnormal uterine bleeding (AUB) and hysteroscopic evidence of CAH in patients with a history of breast cancer and subsequent tamoxifen (TAM) therapy [5]. Outpatient hysteroscopy with biopsy has high diagnostic accuracy of EC and is mandatory in all postmenopausal women with AUB [6].

In women who do not have fertility requirements, the therapy choice for CAH is usually hysterectomy, whereas in younger patients who desire to become pregnant conservative medical or surgical treatments could be offered. Conservative surgical treatment, such as endometrial resection by hysteroscopy, though it is safe and effective, can be suggested to treat focal lesion of CAH [7]. However, in women with diffuse atypical endometrial hyperplasia, high-dose progestin-based medical therapy is an alternate option to regress the lesion. In addition, progestins used are also efficacious in treating early grade endometrial endometrioid adenocarcinoma in women seeking to preserve fertility [1, 2]. Before hormonal therapy, assessment of the extent of malignant lesions, such as depth of myometrial invasion, cervical or parametrial involvement, should be considered. Besides, tumor sizes are easily assessable and useful to estimate the lesion degree and the prognosis of conservative treatment [8]. For advanced EC, progestin therapy results in lower response rates and does not often cure patients.

However, for the high-dose oral progestin treatments, side effects are also accompanied with. A locally high-dose of progestin use that is called levonorgestrel-releasing intrauterine system (LNG-IUS) has been documented for use in the local treatment of endometrial hyperplasia and even CAH or grade-one endometrial cancer, which is considered having treatment effect but not many systemic side effects [2]. In the past few years, we attempted to use LNG-IUS to treat patients with simple and complicated endometrial hyperplasia, and those with CAH and requirement for pregnancy very carefully and with close following-up during clinical practice. The following-up results are promising, and those with CAH treated eventually achieved healthy pregnancy after one or two years of LNG-IUS treatment (unpublished data: the histological regression (glandular atrophy and stroma decidualization) of atypical endometrial hyperplasia using LNG-IUS was observed in the supplemental data in the Supplementary Material available online at http://dx.doi.org/10.1155/2015/758684). A study of 59 patients by Arnes et al. found that LNG-IUS was effective at reducing the occurrence of endometrial polyps [4]. Moreover, a systematic review of literature by Gizzo et al. concluded that LNG-IUS use could decrease the risk of endometrial atypia and polyps in TAM-treated breast cancer survivors [9].

Though it has been reported that progestin could cause atrophy and decidualization of endometrium, the various sources of synthesis progesterone have different biological activities and degrees. Furthermore, the molecular mechanism of progesterone causing these effect remains poorly understood. Therefore, we attempted to study the biological effects of LNG, the main components of LNG-IUS on the primary endometrial cells, through mimicking the local high-dose of LNG on the endometrium, and further the mechanisms that may cause the decidualization of the endometrium.

Connexin 43 (Cx43) is the most widely expressed connexin in the endometrium and is known to be important in a variety of physiological and pathological processes in this tissue [10–13]. Expression of Cx43 has been reported to be regulated by ovarian hormones in the female reproductive tract of rodents. Schlemmer et al. [14] analyzed paraffin-embedded uterine sections from hysterectomy specimens of patients with normal endometrium and from patients diagnosed with grade-one, -two, and -three endometrial cancer and found an inverse correlation between Cx43 expression and tumor grade, which indicated that Cx43 expression may be useful as an adjunctive marker of progression for endometrial carcinoma. In addition, it has been reported that expression of estrogen receptor alpha and progesterone receptors in relation to the expression of Cx26 and Cx43 in endometrial cancer. Disorders of connexin expression and altered distribution pattern occur during endometrial carcinogenesis, and it seems that progesterone receptors could participate in this fact [15]. The expression of Cx43 is positively related to gap junction intercellular communication (GJIC), while the phosphorylation of Cx43 is inversely related to GJIC in endometrial stromal cells. The restoration of GJIC by the induction or transfection of Cx43 was documented to reverse the transformed phenotype of certain cancer types, including those derived from the ovaries, lungs, and cervix [10, 11, 13, 16]. Thus, it appears that the expression and phosphorylation of Cx43 in endometrial cells participate in the maintenance and regulation of endometrial gap junction proteins.

Therefore, we proposed that LNG may suppress the proliferation of endometrial cells via an increase in gap junction permeability in vitro, which is achieved through the upregulation of Cx43 expression and its changed phosphorylated status. In this study, we observed the LNG effects on the cell proliferation and apoptosis of primary human endometrial cells and its role in the changes in the GJIC and Cx43 expression and its phosphorylated status.

## 2. Materials and Methods

### 2.1. Cell Cultures

The primary human endometrial stromal cells (HESCs) and glandular cells (HEGCs) were obtained from patients aged 37–45 years. The patients were undergoing uterectomies as a result of myoma of the uterus and went without hormone treatment for the past three months. The separation of HESCs and HEGCs from eutopic endometrial tissue was performed using the procedure developed by modified methods reported by Ryan et al. [17]. Cells were grown and maintained in DMEM/F12 (Gibco) and supplemented with 10% fetal bovine serum, 2 mm of glutamine, 50 IU/mL penicillin, 50 *μ*g/mL streptomycin, and 1 *μ*g/mL fungizone (complete medium). Cytokeratin and vimentin were detected to determine the successful culture and separation of glandular and stromal cells, respectively, using immunohistochemistry.

### 2.2. Treatment of Cells

LNG and 17*β*-estradiol (E_2_) were diluted in dimethyl sulfoxide (DMSO) with a stock concentration of 1 × 10^−2^ mol/L, and 12-O-tetradecanoylphorbol-13-acetate (TPA) was diluted in DMSO with a stock of 1 *μ*g/mL. These hormones were diluted in accordance with the indicated concentration in the standardized amounts for the experiments. The final concentration of DMSO as a solvent was always less than 0.1%. The treatments of the cells with these hormones were carried out for the time periods and with the concentrations described in our figure legends.

### 2.3. Cell Proliferation Measured by MTT

HESCs and HEGCs were placed in 200 *μ*l of culture media per well in a 96-well plate with a density of 2 × 10^4^ cells/mL (with 8 wells left empty as blank controls) and incubated overnight to allow the cells to attach to the wells. The cells were then treated with 2  *μ*L of LNG in a concentration course manner (1 × 10^−9^, 1 × 10^−5^, 2.5 × 10^−5^, or 5 × 10^−5 ^mol/L) and in a time course (24 h, 48 h, and 72 h), with E_2_ as the opposite control, and DMSO (0.5%) as the negative control. The concentration of LNG displaying an inhibitory effect and that of E_2_ exhibiting a stimulatory effect on cell proliferation (both were 5 × 10^−5 ^mol/L) were adopted as the final treating concentrations during the followed experiments determined according to the preliminary experiments. The concentration of 5 × 10^−5^ mol/L LNG is similar to that in the vivo endometrium in the presence of a mirena with a releasing rate of 20 *μ*g/24 hours of LNG. Afterward, 20 *μ*L of MTT solution was added to each well and incubated (37°C, 5% CO_2_) for 2 to 4 hours until a purple precipitate became visible. When the purple precipitate became visible, 200 *μ*L of DMSO was added and left at room temperature in the dark for 2 hours. The cell growth curve was detected in a *λ* of 597 mm and expressed with an OD value. Finally, the absorbance (optical density) was recorded at 570 nm.

The inhibition rate (IR) was calculated using the following formula:

(1) $$\begin{array}{c} IR=\frac{Control\;\;{OD}_{492}-Experiment\;\;{OD}_{492}}{Control\;\;{OD}_{492}}\times 100\%. \end{array}$$

### 2.4. Apoptosis Measured by Terminal Deoxynucleotidyl Transferase dUTP Nick End Labeling (TUNEL)

HESCs and HEGCs were collected and cultured in six-well plates with glass cover-slips for 48 hours and then treated with 5 × 10^−5 ^mol/L LNG in a time course (24 hs, 48 hs, and 72 hs), with 5 × 10^−5 ^mol/L E_2_ as the opposite control, and DMSO (0.5%) as the negative control. The cover-slips for the cells were fixed with 4% paraformaldehyde, sealed with 3% H_2_O_2_-methanol, and detected using the TUNEL method (cell technology), which was performed in accordance with the recommended protocol. The apoptosis rate (AR) was calculated using the following formula:

(2) $$\begin{array}{c} Apoptosis\;\;rate=\frac{Apoptosis\;cells}{Total\;cells}\times 100\%. \end{array}$$

### 2.5. Analysis of the Cell Cycles Using Propidium Iodide (PI) Staining and Flow Cytometry

HESCs and HEGCs were cultured with a 1 × 10^5^-cell/mL density in 25 mL flasks and then treated with LNG as the experiments above mentioned, with E_2_ as the opposite control and DMSO as the negative control. The cells were then digested and suspended in 0.5 mL of PBS, with a density of 1 × 10^6^/mL, combined with 2 mL of 70% cold ethanol and incubated overnight at −20°C. The cells were also centrifuged at 1200 rpm for 5 min to collect the precipitate. For each sample, 50 *μ*L of RNAase and 450 *μ*L of PI solution were added before incubation for 30 min at 4°C. These suspensions were analyzed by passage through a FACSCalibur flow cytometer (Becton Dickinson); 2 × 10^4^ cells were counted. The percentage of S-phase cells and the cell apoptosis index were computed and analyzed.

### 2.6. Scrape Loading (SL)/Dye Transfer (DT)

The levels of GJIC in both the control and treated cultures were determined using the SL/DT technique (22) with a fluorescent dye called Lucifer Yellow (LY) (Molecular Probes, Eugene, OR). The primary endometrial stromal and glandular cells were cultured in 35 mm plates in the manner described above and treated with LNG or E_2_ in the manner described in Figure 4 legend, with TPA as the control. Scrape loading was performed with a razor blade by applying a cut on the cell monolayer, and the LY was then added to the cells. Then, an incision across the diameter of the clustered regions was made in the presence of a Lucifer Yellow (LY) CH mixture. The cells were washed thoroughly with PBS. The dye was rinsed away after 5 min. The cells were washed three times with PBS and fixed with 4% paraformaldehyde, and the cells stained with LY were detected by fluorescence emission using a reversed fluorescent microscope equipped with either a camera or a laser scanning confocal microscope (LSCM). Cells that received the LY from the scrape-loaded cells were considered to be communicating. A full-automatic photograph analyzing system (KONTRON IBAS2.5, Germany) was used to analyze a 50 × 60 mm^2^ area of dye transfer on both sides of each cut.

### 2.7. Total and Phosphorylated Cx43 (pSer368-Cx43) Measured by Western Blot Analysis

The cells were washed twice with PBS and lysed on ice with a lysis buffer (50 mM Tris-HCl, pH 7.4, 150 mM NaCl, 0.5% Nonidet P-40) containing a complete protease inhibitor cocktail (Roche, Nutley, NJ, USA) and phosphatase inhibitors (1 mM PMSF, 100 mM NaF, and 10 mM Na_3_VO_4_). Then, the lysis of HESCs was measured by western blot analysis with anti-Cx43 antibodies, the total Cx43 expression, or anti-pS368-Cx43 antibodies for S368 phosphorylation of Cx43. Proteins were resolved on a 4–15% gradient SDS-PAGE and transferred to nitrocellulose membranes (Sigma). Immunoblots were incubated with rabbit polyclonal anti-Cx43 antibodies (Sigma) (1 : 80) for the expression of total Cx43, rabbit polyclonal anti-P-Cx43 (S368) (Cell Signaling Technology) for the phosphorylated level of Cx43, followed by incubation with appropriate secondary IgG antibodies conjugated with HRP (Amersham Pharmacia Biotech, Piscataway, NJ, USA). The immunoreactive bands were visualized by the Enhanced Chemiluminescence System (Cell Signaling Technology). Blots were washed, reprobed with anti-*β*-actin (Chemicon) antibodies, and developed in an identical manner for assessing *β*-actin protein levels to ensure even loading.

### 2.8. Total Cx43 and Phosphorylated Cx43 (pSer255-Cx43) Detected by LSCM

HESCs were cultured in six-well plates with cover-slips. When the cells grew to 50% impletion, they were treated with drugs in the manner described in the figure legends. The glass cover-slips containing cells were fixed with 4% paraformaldehyde, sealed with goat serum at room temperature for 30 min, and incubated with anti-mouse Cx43 antibodies (1 : 80) overnight at 4°C, with PBS as the negative control and an unrelated antibody as the control for antibody specificity. After being thoroughly washed with PBS, a FITC-conjugated secondary antibody (1 : 200) was added before incubating the cells for 1 h at 37°C in order to detect total Cx43. For phosphorylatedCx43 (Ser255) detection, the first antibody used was pS255-Cx43 (Santa Gruz) (1 : 80), and the secondary antibody used was Cy3-conjugated IgG (Sigma) (1 : 50). A laser scanning confocal microscope (Zeiss LSCM510 META, Germany) was used to localize different fluorescent-labeled antibodies bound to HESCs. Immunostained cells were scanned with a laser at 488 nm for a green fluorescent of total Cx43 detection or 569–574 nm for a red fluorescent of pS255-Cx43, and optical sections were made either at 0.2 mm or at 1 to 2 mm increments from the top of the cell to the point of contact with the culture plate by means of a stepper motor. Images were obtained from 5–7 scans using Kalman averaging to increase the signal-to-noise ratio; they were viewed on screen and printed via a Sony videoprinter. Fluorescence within a defined region was analyzed by a point counting routine of pixel intensity. Specific counts were added from all sections and then divided by the total pixel areas of the cells to quantify the specific staining of antigens.

### 2.9. Ethics Statement

The institutional review board of Sun Yat-Sen Memorial Hospital, Sun Yat-Sen University specifically approved this study. And the written informed consents from the patients on the endometrium collection have been obtained.

### 2.10. Statistical Analysis

All of the experiments have a minimum of three determinants. The data were expressed as mean ± SD. In some figures, the data are from a representative experiment that was qualitatively similar to the replicate experiments. Statistical significance (*P* < 0.05) was determined with Student's *t*-test (two-tailed) between an individual experimental group and the corresponding control condition set as 100% (one-sample *t*-test).

## 3. Results

### 3.1. LNG Inhibits the Proliferation of Human Endometrial Cells

**Figure 1:**
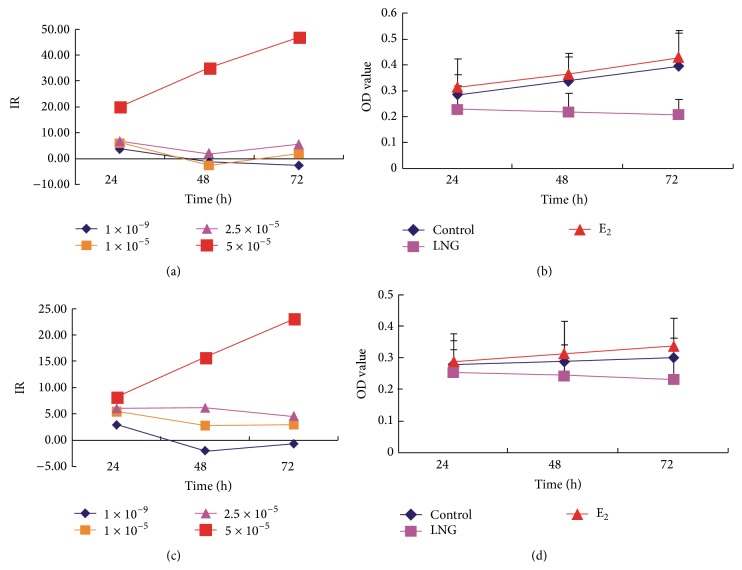
Cell proliferation inhibited by LNG. The MTT cell growth curve was performed to investigate the effects of the treatments on the proliferation of human endometrial stromal cells (HESCs) and glandular cells (HEGCs) treated with LNG, with 5 × 10^−5^ mol/L E_2_ as the opposite control and DMSO (0.5%) as the blank control. (a) The inhibition rate (IR) of the HESCs treated with LNG in various concentrations in a time course manner (24 hs, 48 hs, and 72 hs); (b) MTT cell growth curve of HESCs treated with 5 × 10^−5^ mol/L LNG in a time course manner. (c) IR of the HEGCs treated with LNG in various concentrations in a time course manner; (d) MTT cell growth curve of HEGCs treated with 5 × 10^−5^ mol/L LNG in a time course manner; ^*∗*^ *P* < 0.05; there was a significant difference. Each experiment was repeated for 20 patients.

The cell growth curve study by MTT demonstrated that 5 × 10^−5^ mol/L LNG inhibited the cell proliferation of primary HESCs (Figures 1(a) and 1(b)) significantly over time (24 hs, 48 hs, and 72 hs). In a concentration of 5 × 10^−5 ^mol/L, the inhibitory effect on primary HEGCs (Figures 1(c) and 1(d)) was significant but slightly weaker than that found in HESCs. For the opposite control of E_2_, at the concentration of 2.5 × 10^−5^ mol/L, E_2_ significantly stimulated the proliferation of both HESCs and HEGCs, and this stimulation was stronger for the concentration of 5 × 10^−5 ^mol/L (Figures 1(a) and 1(c)).

### 3.2. LNG Promotes the Apoptosis of Human Endometrial Cells

**Figure 2:**
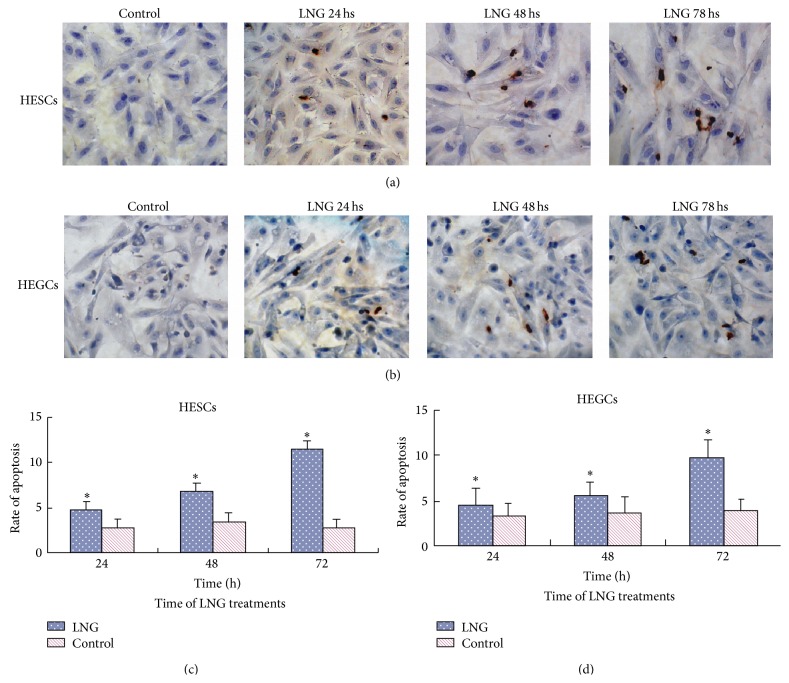
Effects of LNG on endometrial cell apoptosis determined by TUNEL. HESCs (a) or HEGCs (b) were treated in the absence (control) or presence of 5 × 10^−5 ^mol/L LNG for 24 hs, 48 hs, and 72 hs, measured using TUNEL techniques. The brown staining showed the apoptotic body. The histogram represents the mean ± S.D. of the apoptosis rate calculated for HESCs (c) and HEGCs (d) in 6 patients. ^*∗*^ *P* < 0.05; there was a significant difference compared to the control.

**Figure 3:**
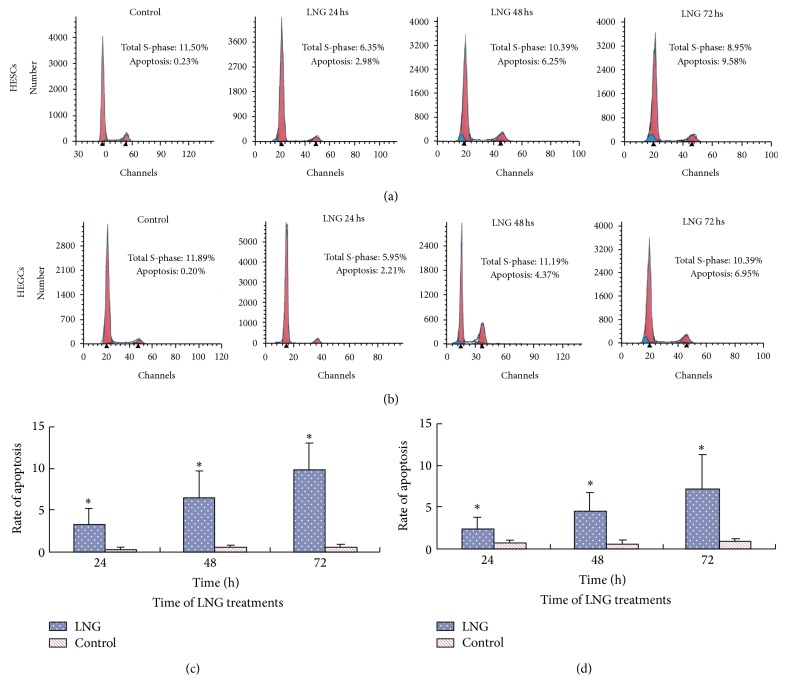
Effects of LNG on the apoptosis of endometrial cells analyzed by propidium iodide (PI) staining and flow cytometry. HESCs (a) and HEGCs (b) were treated in the absence (control) or presence of 5 × 10^−5^ mol/L LNG for 24 hs, 48 hs, and 72 hs. The cell cycle was analyzed by PI staining and flow cytometry, with 2 × 10^4^ cells counted. The histogram represents the mean ± S.D. of the apoptosis rate for HESCs (c) and HEGCs (d) in 6 patients. ^*∗*^ *P* < 0.05; there was a significant difference, compared to the control.

TUNEL showed that the apoptosis rate increased after the treatment with 5 × 10^−5^ mol/L LNG for 24 hs, with a greater increase after 72 hs (*P* < 0.01) in both HESCs (Figures 2(a) and 2(c)) and HEGCs (Figures 2(b) and 2(d)). The apoptosis rate was more significant in HESCs than HEGCs. There was no significant increase in the apoptosis rate after the treatment with E_2_. The propidium iodide (PI) staining and flow cytometry study demonstrated that 5 × 10^−5^ mol/L LNG significantly increased the apoptosis rates of both HESCs (Figures 3(a) and 3(c)) and HEGCs (Figures 3(b) and 3(d)), and the rates increased over time. Moreover, 5 × 10^−5^ mol/L E_2_ had no significant effect on the apoptosis rates in both types of cells.

### 3.3. LNG Enhances GJIC

**Figure 4:**
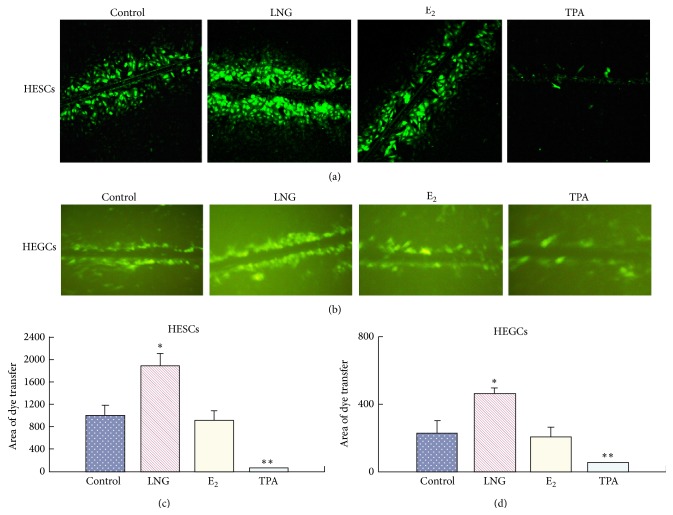
GJIC changes in endometrial cells, measured by scrape loading (SL)/dye transfer (DT). HESCs and HEGCs cells were treated in the absence (control) or presence of 5 × 10^−5^ mol/L LNG or 5 × 10^−5^ mol/L E_2_ for 48 hs or in 10 ng/mL TPA for 2 hs. (a) Lucifer Yellow CH-recipient HESCs, observed by LSCM; (b) Lucifer Yellow CH-recipient HEGCs, observed by a reversed fluorescent microscope; ((c), (d)) The histogram representing the mean ± S.D. of the area of dye transfer for HESCs (c) or HEGCs (d) of 6 patients; ^*∗*^ *P* < 0.05, ^*∗∗*^ *P* < 0.01; there was a significant difference.

To determine the mechanisms responsible for the inhibitory and stimulatory effects on the proliferation and apoptosis of LNG, respectively, and determine which mechanism was related to GJIC changes, we performed SL/DT assays using the gap junction permeable fluorescent dye LY. We found that 5 × 10^−5^ mol/L LNG significantly enhanced the GJIC in the HESCs (Figure 4(a)) compared to the control. The opposite control of the TPA treatment demonstrated that TPA could significantly inhibit GJIC in the HESCs (Figure 4(a)). There was no significant change in the GJIC in the HESCs after 5 × 10^−5^ mol/L E_2_ treatments (Figures 4(a) and 4(c)). The response of GJIC to these drug treatments was similar in the HEGCs (Figures 4(b) and 4(d)).

### 3.4. LNG Enhances the Total Expression of Cx43 but Not the Level of p-S368 Cx43

**Figure 5:**
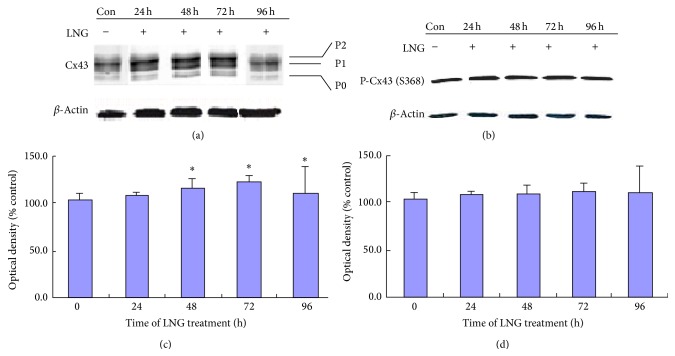
LNG increases connexin 43 protein levels and does not affect the level of p-connexin 43 at the S368 site, as measured by western blot analysis. HESCs were treated with either 5 × 10^−5^ mol/L LNG or the negative control (0.5% DMSO) for 24 hs, 48 hs, 72 hs, and 96 hs; then the protein lysis was measured by western blot (*n* = 11). ((a), (b)) Expression of total connexin 43 (Cx43) (a) and p-S368 Cx43 (b) treated with 5 × 10^−5^ mol/L LNG in a time-dependent manner; ((c), (d)) Comparison of the expression of total Cx43 (c) and p-S368 Cx43 (d) treated with 5 × 10^−5^ mol/L LNG; ^*∗*^ *P* < 0.05; there was a significant difference.

Using western blotting, we investigated the effects of LNG on the protein expression and phosphorylation status of Cx43. Total Cx43 (Figure 5(a)), including nonphosphorylated Cx43 (P0) and phosphorylated Cx43 (P1 and P2), was more strongly expressed after treatment with 5 × 10^−5^ mol/L LNG for 24, 48, and 72 hours in HESCs (Figure 5(c)), with an increased expression over time, while after the LNG treatment for 96 hours, these increased expressions subsided. Furthermore, to test which phosphorylation site was associated with increased levels of P1 and P2, we measured the expression of S368 phosphorylated Cx43 after the treatment with LNG. However, we found that no significant expressive change of p-S368 Cx43 was present (Figures 5(b) and 5(d)).

### 3.5. LNG Promotes the Plasma Expression of Total Cx43 and the Nuclear Translocation of p-S255 Cx43

**Figure 6:**
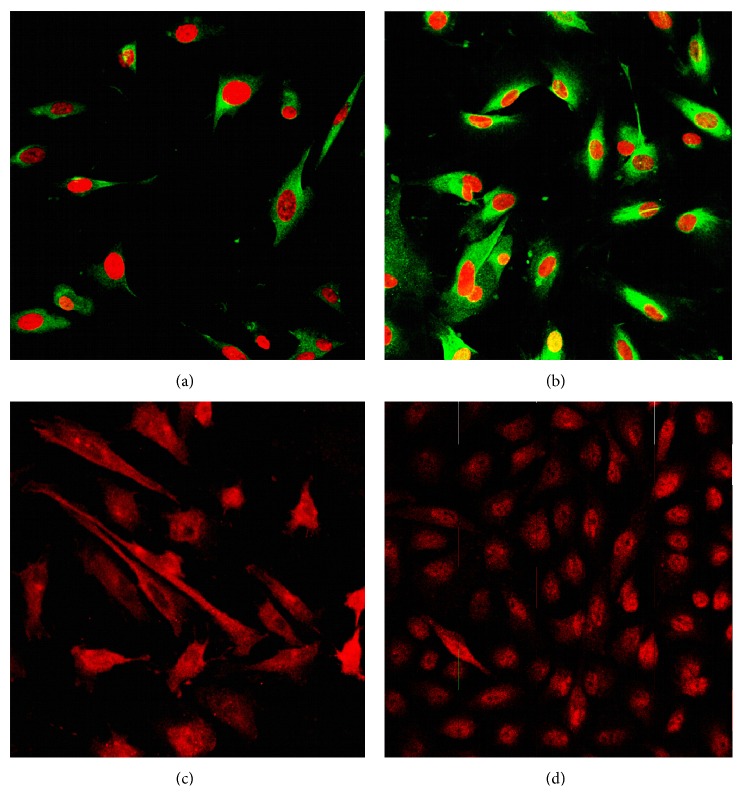
LNG promotes the increased level of total Cx43 in the plasma and the nuclear translocation of p-Cx43 at the S255 site, as measured by LSCM. HESCs were treated with 5 × 10^−5^ mol/L LNG or with the negative control (0.5% DMSO) for 48 hs to detect total Cx43 protein levels or for 5 hs to detect the expression and localization of the p255 Cx43 protein by using LSCM (*n* = 5). ((a), (b)) Expression and localization of total Cx43, treated with 5 × 10^−5^ mol/L LNG or DMSO (control) for 48 hs with PI staining. ((c), (d)) Expression and localization of pS255 Cx43, treated with 5 × 10^−5^ mol/L LNG or DMSO (control) for 5 hs.

We also detected the expression and location of total Cx43 and S255 phosphorylated Cx43 in HESCs using LSCM. Total Cx43 was expressed in both the nuclear compartment and cytoplasm (Figure 6(a)). Moreover, the LSCM confirmed the stronger expression of total Cx43 in the cytoplasm after treatment with 5 × 10^−5^ mol/L LNG for 48 hours (Figure 6(b)). Interestingly, before the LNG treatment, S255 phosphorylated Cx43 was present in the cytoplasm and in some parts of the nuclear compartment, but for the LNG treatment, the p-S255 Cx43 protein was found to be translocated to the nucleus (Figures 6(c) and 6(d)).

## 4. Discussion

In this study, we found that LNG inhibits the cell proliferation and promotes apoptosis in normal human endometrial stromal and glandular cells through the enhancement of GJIC's function in these cells, which may be mediated through the overexpression of total Cx43 and the nuclear translocation of S255 phosphorylated Cx43. This study is the first report on the effect of LNG on the apoptosis of normal human endometrial cells in vitro and on the function and mechanisms of GJIC, of which the abstract was published in a conference proceeding [18].

The inhibition of cell growth and the promotion of cell apoptosis in response to 5 × 10^−5^ mol/L LNG treatment in a time-dependent manner (24, 48, and 72 hours) are consistent with our previous study and other studies suggesting that LNG could lead to the atrophy of the human glandular endometrium in vivo and the histological regression of endometrial hyperplasia and early stage endometrial carcinoma during or at the end of LNG-IUS treatment. Carcinogenesis is a multistage process, and different pathways can lead to cancer. The decreased expression of connexins and alterations in GJIC correlate with tumorigenesis [19]. Connexins in the intracellular (cytoplasmic or nuclear) compartment may control tumor progression, modulating the expression of the genes responsible for cell growth regulation, differentiation and apoptosis, and other functions of cancerous cells. Several studies have suggested that gap junction proteins in endometrial stromal cells play a regulatory role in maintaining normal levels of GJIC in glandular cells [20]. Our study also indicated that LNG could enhance the GJIC function, which is more pronounced in HESCs than HEGCs, suggesting that in addition to connecting the stromal cells, Cx43 is also involved in the inhibition of glandular cells' growth and differentiation. In addition, it has been reported that Cx43 expression is associated with an overexpressed connective tissue growth factor/nephroblastoma (CYR61/CTFG/NOV) family of growth regulators (CNN) [21], such as cyr61, which is an immediate, early gene that encodes a cysteine-rich, heparin-binding protein and a proangiogenic factor that mediates diverse roles in development, cell proliferation, and tumorigenesis. Moreover, Cx43 is originally found synthesized in its nonphosphorylated form and then inserted into the plasma membrane, where it is converted to its phosphorylated forms [22]. A lack of Cx43 expression and the aberrant localization of Cx43 have been associated with a lack of GJIC between tumor cells [23]. Our study found that LNG enhanced the GJIC function via the elevated expression of nonphosphorylated Cx43 (P0). Further investigations of LSCM demonstrated that the expression of Cx43 located in both cytoplasmic and nuclear compartments of the human endometrial stromal cells occurred in a punctuated pattern, which corresponds with the study conducted by McCulloch et al. [24]. After LNG treatment, the level of Cx43 inserted into the plasma membrane increased.

It has been reported that Cx43 can be phosphorylated on at least 14 of the 21 serines and two of the tyrosines in the cytoplasmic tail region (amino acids 245–382). Ser368 is one of the major phosphorylation sites in the carboxyl-terminus of Cx43 by protein kinase C (PKC) [25]. Sáez et al. [26] found that the protein kinase inhibitor, staurosporine, had an inhibitory effect on cell coupling and Cx43 phosphorylation, which is reversed by acute treatment with TPA. Following the study of Richards et al. [27], which showed that the increased amount of Cx43 in S368 phosphorylation reached maximum levels in the skin 24 hours after the wounding and returned to the baseline level by 72 hours, we further measured the effect of LNG on pSer368-Cx43 in a time-dependent manner. In contrast to the effect of TPA, in our study, the expression of pSer368-Cx43 did not change significantly upon LNG treatment, even over a prolonged period of time. On the other hand, Cx43 is reported to be phosphorylated in granulosa cells through a MAPK-dependent mechanism on serines 255, 262, and 279/282 [28] in response to the luteinizing hormone (LH), causing a decrease in the gap junction permeability between the granulosa cells and contributing to the resumption of meiosis in the oocyte [29]. Phosphorylation at these sites is transient and is no longer phosphorylated by 5 hours after the LH treatment [28]. On the basis of these characteristics of pS255 Cx43 over time, we detected the serines 255 phosphorylation of Cx43 upon LNG treatment using LCSM. In our study, more cells expressed the Ser255 phosphorylated Cx43 and the translocation of pS255 Cx43 from the plasma to the nuclear compartment in human endometrial stromal cells. The increased phosphorylation of Cx43 mediated by two serine/threonine protein kinase families, protein kinase C (PKC) [25] and MAPK [28], was reported to be causally linked with the disruption of GJIC. Although raloxifene could also work on cellular enhanced attachment and migration, it plays a role by interfering the recruitment of the Ga13/RhoA/ROCK/moesin cascade [30] and as a selective estrogen receptor modulator. Therefore, LNG may have a completely different molecular mechanism from raloxifene, working as a potent progesterone on the progesterone receptor. Based on this theory, our observations led to the demonstration that LNG increases the gap junction permeability in endometrial stromal cells via the translocation of Ser255 phosphorylated Cx43 from the plasma to nuclear compartment, consistent with the increased, nonphosphorylated Cx43 levels in the plasma.

## 5. Conclusions

In conclusion, LNG could inhibit the cell proliferation of and promotes apoptosis in human endometrial stromal and glandular cells though increasing the gap junction permeability in vitro study. The complete connection among the stromal cells is important in the normal growth of endometrial cells. The stronger GJIC function may be achieved by the upregulation of Cx43 expression and the translocation of serine 255 phosphorylated Cx43 from the plasma to the nuclear compartment. The data suggest a novel mechanism by which LNG can influence endometrial cell biology, which provides a theory basis for the use of LNG-IUS in the conservative treatment of endometrial cancer of early stage as an optional fertility sparing approach [30].

## Supplementary Material

The histology of the endometrium in a 30-year old woman with endometrial intraepithelial neoplasms (EIN) and eventually achieving a live birth showed the high-grade atypical endometrial hyperplasia (EH) of the glandular cells with relatively less stromal cells was observed, before the treatment of LNG-IUS (A); the high-grade atypical EH had inverted to the low to medium-grade atypical EH with partly stromal decidualization, after the treatment of LNG-IUS for 6 months; and was reverted to the normal endometrium with atrophy of endometrial glandular cells and obvious stromal decidualization, after the treatment of LNG-IUS for 12 months.

## List of Abbreviations

GJIC: Gap junction intercellular communication
Cx: Connexin
LNG-IUS: Levonorgestrel-releasing intrauterine system
HESCs: Human endometrial stromal cells
HEGCs: Human endometrial glandular cells
EC: Endometrial cancer
PCOS: Polycystic ovary syndrome
CAH: Endometrial complex atypical hyperplasia
ER alpha: Estrogen receptor alpha
PRs: Progesterone receptors
AR: Apoptosis rate
LSCM: Laser scanning confocal microscope.
