# Supplementary figures and images for: Levonorgestrel Inhibits Human Endometrial Cell Proliferation through the Upregulation of Gap Junctional Intercellular Communication via the Nuclear Translocation of Ser255 Phosphorylated Cx43
Source: Biomed Res Int. 2015 Jun 16;2015:758684. doi: 10.1155/2015/758684 (PMC4486311; doi:10.1155/2015/758684)

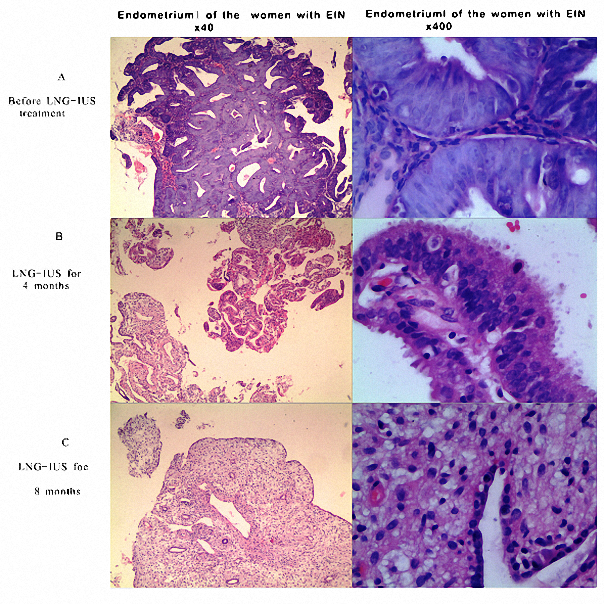

Supplement: Supplementary file 1 — The histology of the endometrium in a 30-year old woman with endometrial intraepithelial neoplasms (EIN) and eventually achieving a live birth showed the high-grade atypical endometrial hyperplasia (EH) of the glandular cells with relatively less stromal cells was observed, before the treatment of LNG-IUS (A); the high-grade atypical EH had inverted to the low to medium-grade atypical EH with partly stromal decidualization, after the treatment of LNG-IUS for 6 months; and was reverted to the normal endometrium with atrophy of endometrial glandular cells and obvious stromal decidualization, after the treatment of LNG-IUS for 12 months. [file 758684.f1.tif]
